# Diesel exhaust particles modify natural killer cell function and cytokine release

**DOI:** 10.1186/1743-8977-10-16

**Published:** 2013-04-24

**Authors:** Loretta Müller, Claire VE Chehrazi, Michael W Henderson, Terry L Noah, Ilona Jaspers

**Affiliations:** 1 Department of Pediatrics, University of North Carolina at Chapel Hill, Chapel Hill, NC, USA; 2 Center for Environmental Medicine, Asthma and Lung Biology, University of North Carolina at Chapel Hill, Chapel Hill, NC, USA; 3 Biological and Biomedical Sciences Program, University of North Carolina at Chapel Hill, Chapel Hill, NC, USA; 4 Curriculum in Toxicology, University of North Carolina at Chapel Hill, Chapel Hill, NC, USA

**Keywords:** Air pollution, NK cells, Diesel exhaust, Cell-mediated cytotoxicity, Polyriboinosinic acid-polyribocytidylic acid

## Abstract

**Background:**

Natural killer (NK) cells are an important lymphocyte population in the nasal mucosa and play important roles in linking the innate and the adaptive immune response. Their two main functions are direct cell-mediated cytotoxicity and the release of cytokines. They are important during viral infections and cancer. Due to their location in the nasal mucosa, NK cells are likely exposed to inhaled pollutants, such as diesel exhaust. Whether and how exposure to diesel exhaust particles (DEP) affects NK cell function in the context of viral infections has not been investigated.

**Methods:**

NK cells were isolated from peripheral blood obtained from normal healthy volunteers and subsequently stimulated with the viral mimetic polyinosinic:polycytidylic acid (pI:C), DEP, or pI:C+DEP for 18 hours. NK cells were subsequently analyzed for changes in surface marker expression, cytokine production, gene expression changes, and cytotoxic function using flow cytometry, ELISA, qRT-PCR, and cell-mediated cytotoxicity assay, respectively.

**Results:**

Stimulation of NK cells with pI:C and pI:C+DEP, but not DEP alone, increased the release of IL-1β, IL-2, IL-4, IL-8, IL-10, IL-12p70, IFN-γ and TNF-α. As compared to pI:C alone or pI:C+DEP, the release of IL-1β, IL-8 and TNF-α was significantly lower after DEP stimulation alone. Stimulation with pI:C alone increased the gene and protein expression of granzyme B and perforin, which was completely blunted by adding DEP. Addition of DEP further reduced CD16 expression in pI:C stimulated cells. Similarly, cell-mediated cytotoxicity was significantly reduced by the addition of DEP.

**Conclusions:**

In the context of viral infection, DEP potentially reduces NK cells' ability to kill virus-infected host cells, in spite of normal cytokine levels, and this may increase susceptibility to viral infections . This reduction in the potential ability of NK cells to kill virus-infected host cells may increase the susceptibility to viral infections after DEP exposure.

## Background

Natural killer (NK) cells make up about 10% of the lung lymphocyte population [1] and are an important immune cell population in the human nasal mucosa [2]. NK cells are an important first line of defense against invading microbes, particularly viruses [1], and play an important role in linking the innate and the adaptive immune response [3]. Their two main effector functions are 1) direct killing of infected cells through release of perforin and granzyme B; and 2) secretion of cytokines to stimulate both innate and adaptive immunity [4]. Based on effector function, NK cells are divided into two major subsets: more cytotoxic NK cells (CD56^dim^CD16^+^) and more cytokine-secreting NK cells (CD56^bright^CD16^dim/-^) [5]. CXCR3 expression on the surface of NK cells promotes chemotaxis to sites of infection through the release of IP-10 by other cells including epithelial cells [6]. NKG2D is an activating NK cell surface receptor that recognizes ligands, such as MHC class I polypeptide-related sequence (MIC) A and B and UL16-binding proteins (ULPBs), displayed on the surfaces of stressed cells, thereby preventing non-specific killing by bringing NK cells into close proximity with their targets [7]. It has previously been shown that CXCR3 and NKG2D undergo upregulation in response to viral infection [2,8]. In addition, hemagglutinins derived from influenza and parainfluenza bind to the activating receptor NKp46, which is involved in the lysis of MHC class 1-negative cells and in tumor recognition [9,10].

Few studies have evaluated the impact of air pollution on NK cells. Williams and colleagues found diminished NK cell cytotoxicity in postmenopausal, overweight women living and exercising near major roadways [11] and diesel exhaust (DE) was shown to suppress the *in vivo* IFN-γ production by NK cells in mice [12]. Besides particulate air pollution, exposure to ozone has been shown to reduce the cytotoxicity and IFN-γ production by NK cells [13,14]. We and others have previously demonstrated that exposure to DE increases the susceptibility of epithelium to viral infection [15,16], including influenza [17,18].

Steadily increasing urbanization, significant numbers of diesel engine-driven vehicles on the roads, and high peaks of particulate matter concentrations in urban areas raise the question about the effects of diesel exhaust particles (DEP) on the immune system. Clarification of the mechanistic interactions between air pollution and the immune system may provide insight into prevention of air pollution-related diseases. Since NK cells play crucial roles in fighting viral infections [1,3,19], we investigated the effect of DE particles (DEP) on NK cell activity and function. In this study, we evaluated the effects of DEP on peripheral blood NK cells from healthy, nonsmoking, non-asthmatic volunteers in the setting of stimulation with a mimetic of virus-derived dsRNA [polyriboinosinic acid-polyribocytidylic acid (pI:C)]. Our data indicate that exposure DEP decreases markers of cytotoxic NK cells and functionally suppresses cell-mediated cytotoxicity.

## Results

### Cytokine release

Stimulation with pI:C significantly increased release of all cytokines (IL-1β, IL-2, IL-4, IL-8, IL-10, IL-12p70, IFN-γ, or TNF-α) tested other than IL-5 and IL-13 (Table 1). DEP alone had little impact on cytokine release, other than a modest increase of IL-1β, IL8, and TNF-α. In the presence of DEP, pI:C-induced increases were blunted somewhat for most cytokines, though this change was not statistically significant except for IL-4.

**Table 1:** Cytokine release of NK cells after stimulation with pI:C, DEP and pI:C+DEP

	**pI:C**	**DEP**	**pI:C+DEP**
IL-1β	*193.9±111	^#^6.09±5.0	*111.7±61.6
IL-2	*2.11±0.44	1.13±0.16	*1.191±0.38
IL-4^£^	*5.99±2.35	1.58±0.55	*2.55±0.66
IL-5	108.6±104	1.56±0.62	6.26±3.21
IL-8	*10.2±3.08	^#^1.53±0.65	*9.53±2.87
IL-10	*21.0±9.4	5.01±2.8	*30.6±19.4
IL-12p70	*142±109	56.7±53.6	*82.0±60.7
IL-13	15.1±6.75	1.82±0.59	8.81±4.4
IFN-γ	*844±414	496±495	*520±241
TNF-α	*37.1±14.6	^#^3.08±1.87	*26.4±8.81

Data are presented as fold induction over control and shown as mean ± SEM. *^,#,£^p<0.05 (n=9).

*significant different from untreated control.

^#^ significant different from pI:C and pI:C + DEP.

^£^ significant difference between pI:C and pI:C+DEP.

### Granzyme B and perforin expression

**Figure 1:**
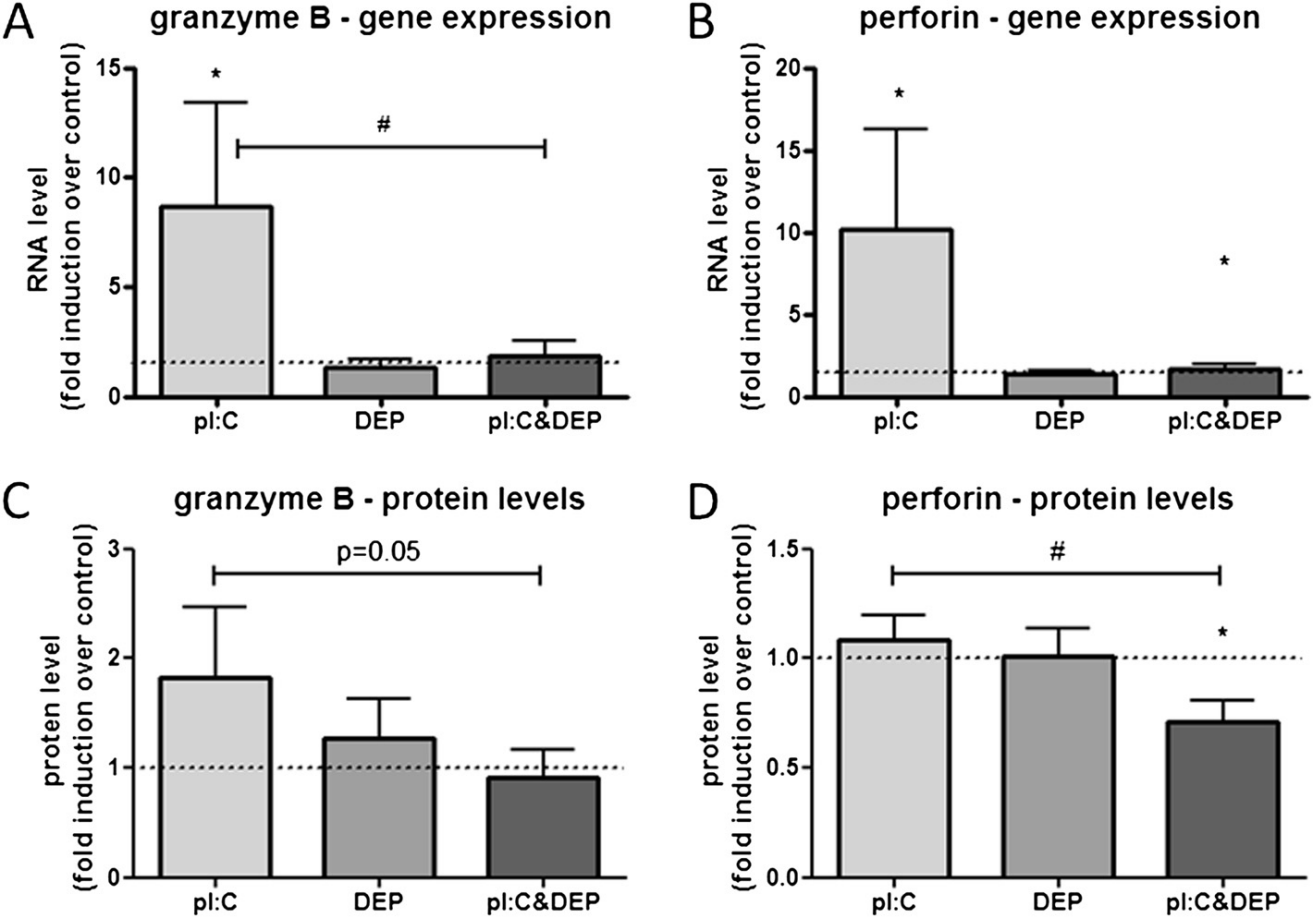
**RNA levels of cytotoxicity related factors in NK cells after stimulation with pI:C, DEP and pI:C+DEP.** (**A**) Gene expression of granzyme B and (**B**) gene expression of perforin expressed as fold induction over untreated control. (**C**) Protein levels of granzyme B and (**D**) protein levels of perforin expressed as fold induction over untreated control. Data are presented as mean±SEM. *significantly different from the untreated control, ^#^ significantly different between each other (both tested with a Wilcoxon signed rank test, *^,#^p<0.05, n=9).

Expression and release of granzyme B and perforin are important during NK cell-mediated lysing of target cells. The RNA levels of granzyme B and perforin were both affected by pI:C and DEP (Figure 1). pI:C alone significantly increased the expression of granzyme B and perforin compared to the vehicle control. DEP alone did not affect the RNA level of either granzyme B or perforin. However, pI:C-induced expression of granzyme B was significantly reduced by the addition of DEP. Similarly, granzyme B and perforin protein levels were significantly reduced in samples exposed to pI:C&DEP compared to pI:C alone.

### NK cell phenotype

NK cells were analyzed for surface marker expression associated with NK cell activation (NKG2D and NKp46) or cytotoxic potential (CD16) using flow cytometry. Forward and side scatter properties revealed a small cluster of lymphocytes and gating on CD3 and CD56 demonstrated a significant majority of lymphocytes to be CD3^-^CD56^+^ NK cells and not NKT cells.

**Figure 2:**
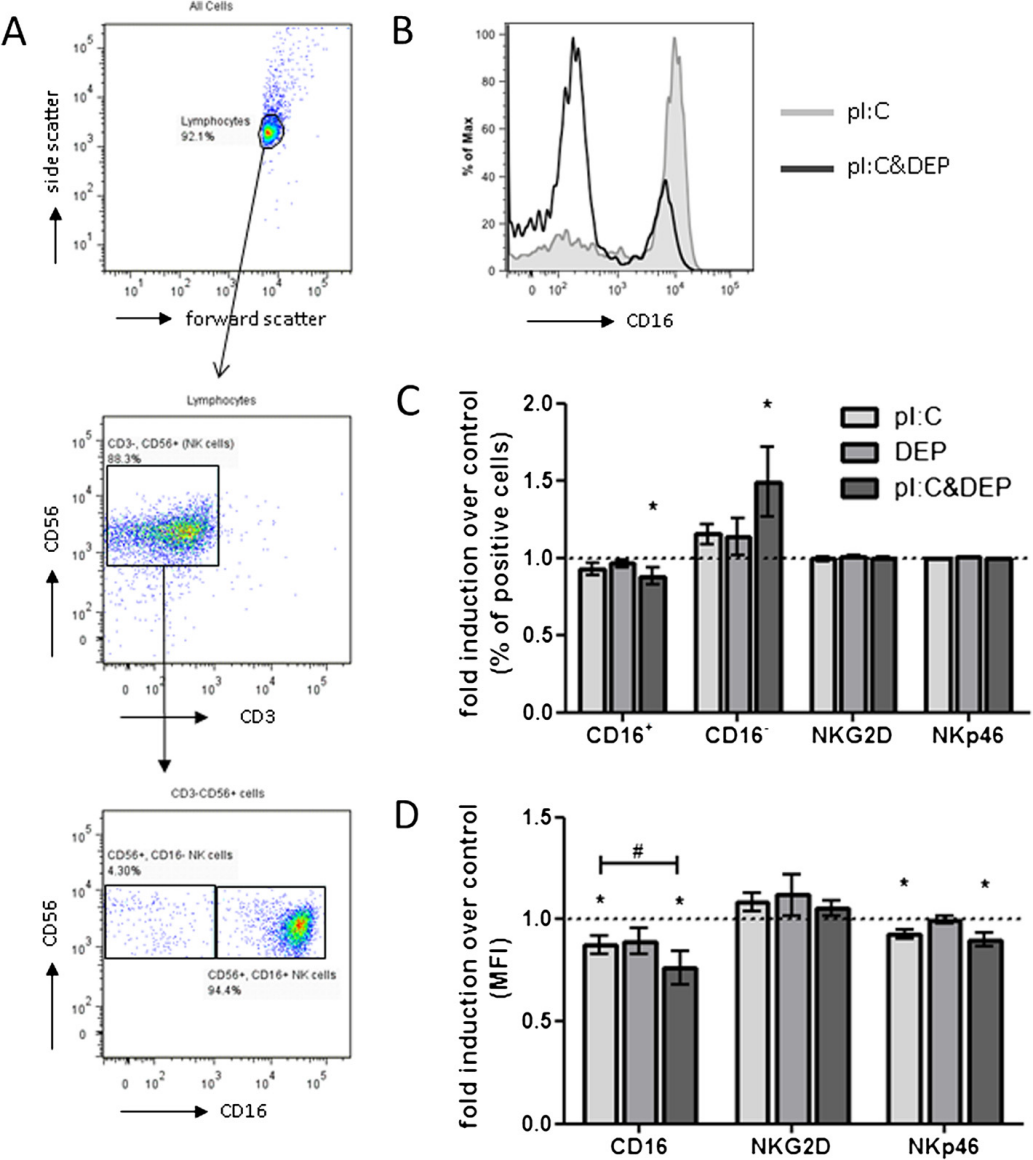
**NK Cell phenotypes assessed by flow cytometry after exposure to pI:C, DEP and pI:C+DEP.** (**A**) Gating strategy for the phenotype analysis. (**B**) Representative histogram showing that CD16 expression decreased after p:IC+DEP exposure compared to pI:C alone and the subset of CD16- NK cells increased. (**C**&**D**) Surface markers presented as fold induction over untreated control of percent of positive total NK cells (**C**) or mean fluorescecne intensity (MFI) (**D**). Data are presented as mean±SEM. *significant different from the untreated control, ^#^ significant different between each other (both tested with a Wilcoxon signed rank test, *^,#^p<0.05, n=9).

Expression of CD16, a marker of the cytotoxic activity of NK cells, was reduced in cells stimulated with pI:C+DEP. While pI:C alone decreased the mean fluorescence intensity (MFI) of CD16, addition of DEP further reduced the expression of CD16 in these cells. Conversely, the percentage of CD16^-^ NK cells was increased by pI:C+DEP (Figure 2).

Neither the percentage of NKG2D^+^ cells nor the MFI of NKG2D was changed in either of the different treatment groups. Stimulation with pI:C and pI:C+DEP reduced the MFI of NKp46, but did not change the percentage of NKp46^+^ cells. Stimulation with DEP alone showed no effects on the NKp46 expression on NK cells (Figure 2).

### Cell-mediated cytotoxicity

**Figure 3:**
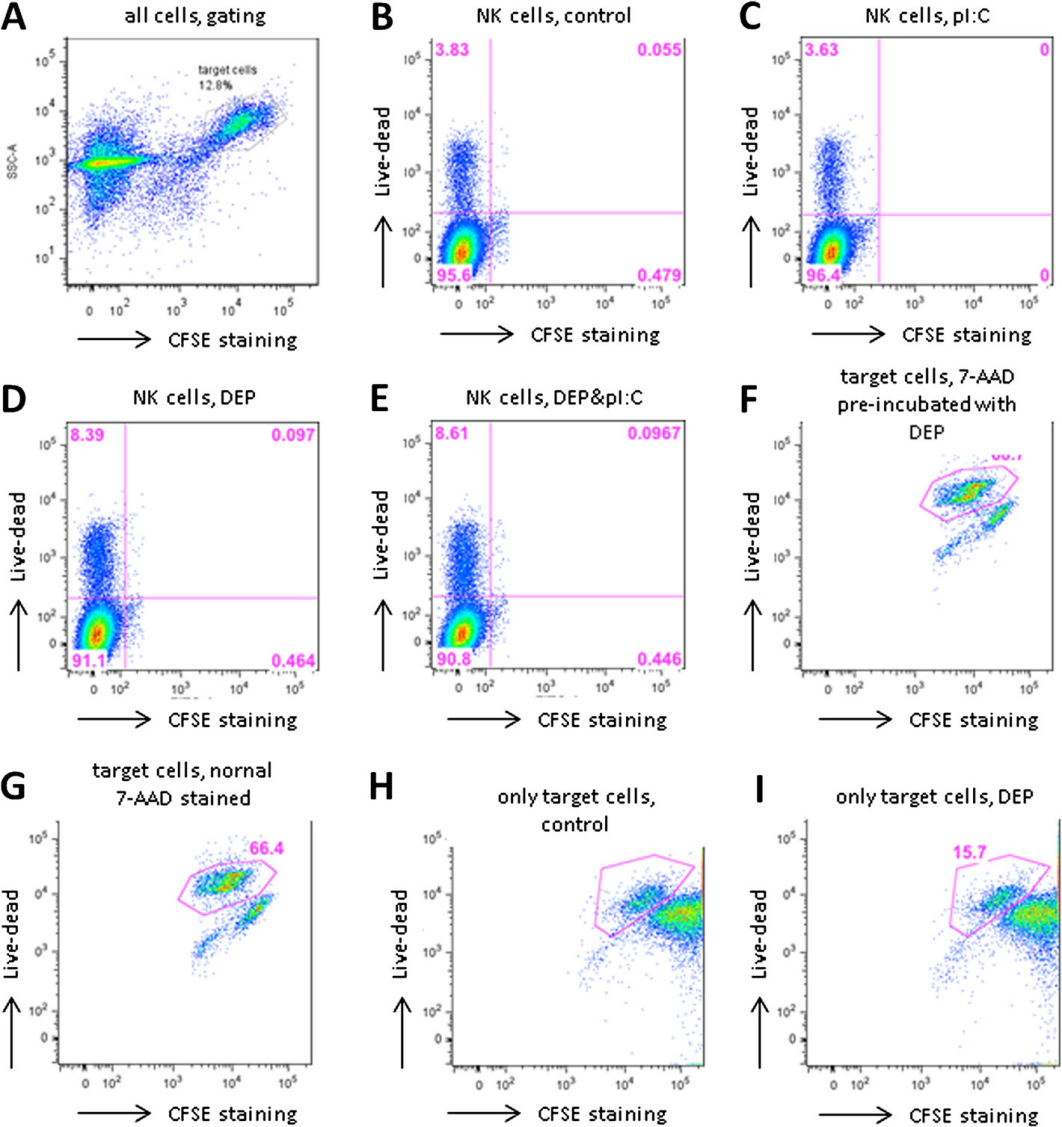
**Controls for the cell-mediated cytotoxic potential of NK.** (**A**) Representative dot plot of the NK-target cell mixture. Target cells were gated based on side scatter (SSC) properties and CFSE staining. (**B**-**E**) NK cell viability is not affected signifcantly by treatment DEP. Only cells gated for NK cells (based on SSC and negative CFSE staining) are shown. (**F**&**G**) The live-dead dye 7-AAD is not adsorbed by DEP. (**F**) 7-AAD pre-incubated with DEP for 15 min before staining a control sample of NK-target cell mixture after 4 hrs of incubation. (**G**) Control sample stained with 7-AAD normally after 4 hrs of incubation. (**H**&**I**) DEP do not kill target cells. (**H**) Target cells only stained with 7-AAD. (**I**) Target cells only incubated with DEP and stained with 7-AAD.

Optimization experiments yielded the conditions chosen here to determine the regulation of cell-mediated cytotoxicity (3 hrs target-effector cell incubation, same media for target and NK cells, NK:target cell ratio of 5:1). Controls were run in order to test potential interactions of the stimuli with the target cells or the dye. The 7-AAD dye was not adsorbed by the DEP (Figure 3F&G), DEP did not kill target cells (Figure 3H&I) and only few NK cells were killed during the assay (Figure 3B-E). The average level of baseline cell-mediated cytotoxicity was 53.3% (SEM 4.4) killing of target cells, with a range of 22.2 to 82.6%. As a positive control NK cells were stimulated with IL-12, which yielded a 1.7 fold (SEM=0.24) induction compared to the control.

**Figure 4:**
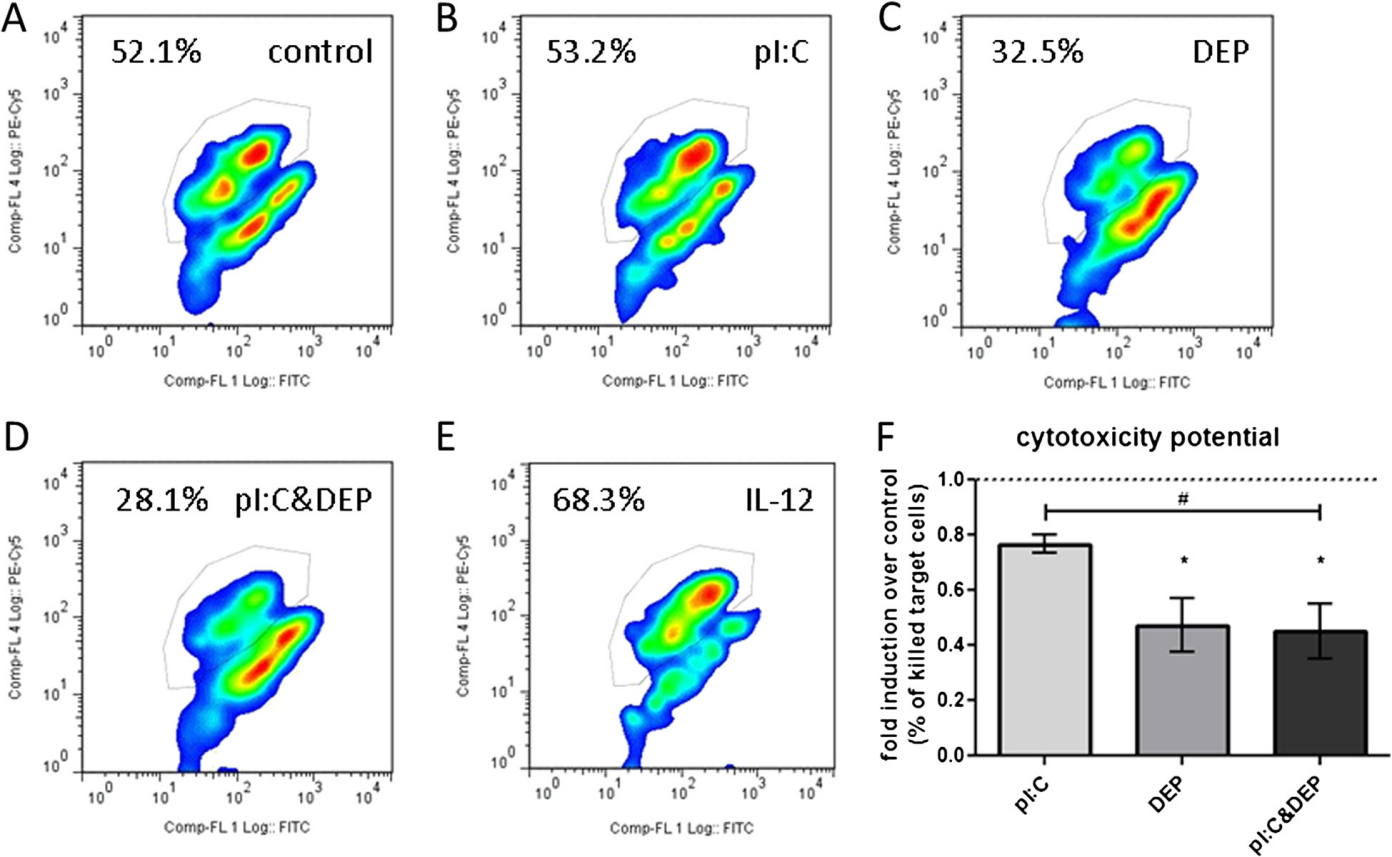
**Cell-mediated cytotoxicity potential of NK cells exposed to pI:C, DEP or pI:C+DEP.** Results were assessed as percentage of killed target cells (K562 cancer cell line) and are expressed as fold induction over untreated NK cells. (**A**-**E**) Representative dot plots of all four conditions plus IL-12 stimulated NK cells as positive control to enhance cytotoxicity. (**F**) Summary of 5 individual experiments. Data are presented as mean±SEM. *significantly different from control, ^#^significantly different between each other, *^,#^p<0.05.

Stimulation with DEP alone significantly reduced the cytotoxicity potential as compared to control (Figure 4). Stimulation with pI:C alone did not significantly affect NK cell mediated cytotoxicity, but addition of DEP (pI:C+DEP) significantly reduced cytotoxicity as compared to the control or pI:C alone.

## Discussion

Exposure to particulate matter (PM) has been associated with increased mortality and hospital admissions due to cardiovascular and respiratory disease [20], including influenza-related mortality [21]. We and others have previously shown that exposure to DE increases the susceptibility for viral infections [15,16], such as influenza [17,18]. NK cells play important roles during antiviral host defense response by killing virus infected host cells and by initiating an immune response via the release of cytokines [3,4]. Despite the important roles NK cells play in respiratory immunology and host defense, the potential impact of air pollutants, such as DE, on NK cells function has not been thoroughly investigated. Our results indicate that in the context of stimulation with the viral mimetic pI:C, DEP decreases markers of cytotoxic function, such as CD16 expression as well as the expression of granzyme B and perforin, and suppresses the ability of NK cells to kill target cells. Thus, impaired antiviral host defense responses seen after exposure to DEP may be related to the direct effects these pollutants have on NK cell function.

In addition to producing cytokines [3], direct killing of (virus-) infected cells and transformed tumor cells are functions of NK cells [4]. Reducing the cytotoxic potential of NK cells by exposure to environmental stressor, such as particulate matter, could affect the ability to fight viral infections. We have recently demonstrated that in nasal passages of human volunteers, NK cells comprise a significant portion of the mucosal lymphocytes [2].

These studies also demonstrated that in the context of viral infection, markers of NK cell cytotoxicity were reduced in smokers as compared to non-smokers [2], which was associated with increased viral replication in a similar cohort [22]. The data presented here demonstrate that DEP suppresses the cytotoxic potential of NK cells. Our previous study conducted in human volunteers exposed to DE prior to inoculation with live attenuated influenza virus (LAIV) indicated that DE exposure increases quantity of virus in nasal secretions [23]. DE-induced suppression of NK cell cytotoxicity, as shown here, could lead to reduced killing of virus-infected host cells and facilitate enhanced virus replication in the nasal mucosa.

NK cells use three different mechanisms to carry out their cytotoxic effector function: 1) Release of cytotoxic mediators (granzyme B and perforin), 2) Fas-Fas ligand mediated pathway, and 3) cytokine dependent pathway (cross-linking of TNF and TNF receptor) [24]. Our data show decreased gene expression as well as protein levels of granzyme B and perforin due to DEP exposure (Figure 1), which correlated with a reduced ability of NK cells to kill target cells after DEP exposure (Figure 4). In addition, our data presented in Table 1 indicate no significant effects of DEP on pI:C-induced TNF-α release by NK cells, suggesting that the TNF/TNFR (mechanisms 3) does not play an important role in mediating the DEP-induced suppression of the NK cell cytotoxicity. We cannot completely exclude Fas-Fas ligand mediated pathway playing a role in the DEP-induced suppression of NK cell cytotoxicity. Even though 7-AAD used in the cytotoxicity assay here stains target cells after entering dead or damaged cells by entering through pores in the cell membrane, thus favoring necrotic cells, we cannot rule out apoptotic cell death of target cells in the cell-mediated cytotoxicity assay used here.

Exposure to DEP could affect the granzyme B/perforin mediated cytotoxicity at two levels. First, the expression of granzyme B and perforin can be regulated transcriptionally. Various signaling pathways and transcription factors can be involved, such as Ikaros, core-binding factor (CBF), activator protein (AP) -1, nuclear factor kappa B (NF-κB), janus kinase (JAK) 1, signal transducer and activator of transcription (STAT) 3 and 5 [25-27]. Studies conducted in bronchial epithelial cells have demonstrated that exposure to DEP activates AP-1, NF-kB, and STAT3 [28,29]. Second, the lysis of granzyme B and perforin depends on the intracellular movement of granzyme B and perforin containing vesicles to the target cell. Phosphatidylinositide 3-kinases (PI3K) plays a pivotal role in regulating this intracellular movement [30]. PI3K and NFκB are known to be affected by DEP exposure [31] and may be involved in the DEP-induced suppression of NK cell cytotoxicity. Thus, DEP may impair NK cell cytotoxicity, and more specifically granzyme B and perforin formation and release both transcriptionally and post-translationally.

In addition to cell-mediated cytotoxicity, NK cells play important roles during innate immune responses and provide a bridge to adaptive immunity by being a significant source of cytokines [3]. In particular, NK cells have been shown to be important sources for IFN-γ, TNF-α, IL-1, IL-2, IL-4, IL-5, IL-10, IL-13, IL-8, and IL-12 [32-35]. We tested the production of these cytokines by NK cells and demonstrated that most were enhanced by stimulation with pI:C alone or pI:C+DEP, though levels for IL-5 and IL-13 did not reach statistical significance. Stimulation with DEP alone did not affect any of the cytokines measured here. However, addition of DEP to the pI:C stimulation (pI:C+DEP) tended to reduce the release of most of the cytokines as compared to pI:C alone, and this reached statistical significance for IL-4. The release of cytokines by NK cells is important for linking the innate and adaptive immune response via recruiting and activating other immune cells [3]. NK cell-derived cytokines activate dendritic cells [36,37] and help to shape T cell responses after NK cells have migrated to secondary lymphoid compartments [38]. The suppression of cytokine production of NK cells by DEP could result in an overall reduced or modified immune response.

Maturation and activation of NK cells *in vivo* depends on a complex microenvironment which defines the activation status of NK cells via soluble mediators as well as direct receptor-ligand interactions [6,39-42]. *In vivo* various cytokines with activation potential for NK cells [4,43-46] may be released by other cells types (e.g. dendritic cells, macrophages, T cells, and epithelial cells) and can change the activity and functionality of NK cells. In our study, isolated peripheral blood NK cell responses in the context of a viral mimic were investigated, which is a potential limitation of our data. However, since the cytotoxicity potential of NK cells exposed to DEP *in vitro* was heavily impaired and corresponds with our findings in human volunteers exposed to DE *in vivo*[23], the effects shown here may have relevance for respiratory NK cells *in vivo*. Another potential limitation of our study is the use of the viral mimetic pI:C, which represents a model of double-stranded RNA (dsRNA) formed during viral replication. dsRNA is generated during infections with positive single stranded RNA (ssRNA) viruses (such as rhinovirus) and to a lower extent during infection with negative ssRNA viruses (such as influenza virus and RSV) [47]. While this is a potential limitation, it also allowed us to examine more general mechanisms by which DEP affects NK cell function and avoid factors introduced by active infection with a replicative virus.

In addition to respiratory virus infections, NK cells also play a role in several pulmonary bacterial and fungal infections (as reviewed by [1]). For example, NK cells are important for the clearance of *Pseudomonas aeruginosa* from the lung [48]. Exposure of mice to DE decreased bacterial clearance in exacerbated pulmonary pathology associated with *Pseudomonas aeruginosa* infection [49]. The authors from this study concluded that exposure to DE increases susceptibility to *Pseudomonas aeruginosa* infection, resulting in increased epithelial damage. Similarly, NK cells play important roles during *Mycobacteria tuberculosis* infections by inducing apoptosis of infected monocytes/macrophages (reviewed by [1]). Exposure of mice to DEP prior to infection with *Bacillus Calmette-Guerrin*, a mouse model of *M. tuberculosis* in mice, showed that DEP impaired clearance of the pathogen [50]. These studies also demonstrated that the number of NK cells or ability to produce IFN-γ was not impaired by DEP, but attributed the reduced clearance to lower macrophage responsiveness to IFN-γ. Neither one of these studies examined whether exposure to DEP affected the ability of NK cell to directly kill the pathogen or induce cell-mediated cytotoxicity in infected cells. It is possible that reduced NK cell-mediated cytotoxicity after DEP exposure may have contributed to the effects of DEP on *Pseudomonas aeruginosa* or *M. tuberculosis* infections.

## Conclusions

We showed that exposure to DEP reduced expression of the cytotoxic NK cell surface marker CD16, gene and protein expression of granzyme B and perforin, and the ability to kill target cells. Considering the role NK cells play during respiratory virus and bacterial infections, DEP-induced reduction of the cytotoxic potential in NK cells may play a role in the increased susceptibility to viral and bacterial infections seen after exposure to particulate matter and more specifically, DE.

## Methods

### Natural killer (NK) cell isolation

Blood was drawn from each subject (Table 2) using a protocol approved by the University of North Carolina School of Medicine Institutional Review Board for Biomedical Research. In addition, written informed consent was provided by each study participant. For flow cytometry surface marker experiments and protein analysis peripheral blood mononuclear cells were enriched for NK cells as previously described [2] using the RosetteSep® Human NK Cell Enrichment Cocktail (Stemcell, Vancouver, British Columbia. For the cytotoxicity assay, NK cells were isolated from PBMCs using Dynabeads NK isolation kits (Dynabeads Untouched Human NK Cells, Invitrogen) according to the supplier’s instruction as previously described [13] NK cells were resuspended in RPMI 1640 with L-Glutamine (Gibco, Invitrogen, Grand Island, NY, USA) with 10% FBS (Gibco) and 1% Pen/Strep (Gibco).

**Table 2:** Characteristics of the healthy, nonsmoking volunteers (without previous viral infection)

**Age (years)**	**Gender**	**Race**	**BMI (kg/m^2^)**
20	f	African-American	33.5
22	m	Caucasian	25.8
22	f	Caucasian	23.1
23	m	Caucasian	21.9
23	f	Caucasian	28.5
23	m	Caucasian	N/A
24	f	N/A	N/A
24	m	Caucasian	25.8
27	f	African-American	21.5
27	m	Caucasian	25.1
28	f	Caucasian	23.2
28	m	Caucasian	27.6
31	f	African-American	36.1
32	m	Caucasian	23
35	m	Caucasian	23
36	f	Caucasian	25
**mean: 26.6±4.8**	**Female /male**	**African-American /Caucasian**	**mean: 25.9±4.3**
**range: 20-36**	**8/8**	**3/12**	**range: 21.5-36.1**

*BMI* = body mass index, *N/A* = not available.

### NK cell stimulation

NK cells (at a concentration of 10^6^cells/ml, using 1×10^5^ cells/condition for flow cytometry analysis of surface marker expression, PCR and protein analysis and 2×10^5^ cells/condition for the cell-mediated cytotoxicity assay) were incubated at 37°C for 18–20 hours in RPMI complete medium with vehicle, pI:C (Sigma, St. Louis, MO, USA, at 10 μg/mL), diesel exhaust particulates (DEP, 10 μg/ml) or pI:C+DEP. DEP were generated as previously described with a 30-kilowatt (kW) four-cylinder Deutz BF4M1008 diesel engine connected to a 22.3-kW Saylor Bell air compressor to provide a load [28,51]. DEP was stored in Hank’s Buffered Salt Solution (HBSS) at −20°C. To achieve a homogenous particle suspension, after thawing, DEP was sonicated for 90 seconds immediately prior to use. Following stimulation for surface marker and cytokine analysis, samples were centrifuged at 500 g, 4°C for 5 minutes to pellet the cells. Supernatants were decanted and stored at −20°C for subsequent analysis of mediators.

### Flow cytometry analysis

NK cells were resuspended and washed in 1 mL flow staining buffer (1% heat-inactivated FBS and 0.09% sodium azide in Dulbecco’s phosphate buffered saline without magnesium or calcium), then stained in the dark for 20 minutes at room temperature with CD3-APC/Cy7 (Biolegend, Tokyo, Japan), CD56-PE (BD Biosciences, Franklin Lake, NJ), CD16-FITC (Beckman-Coulter, Brea, CA), CXCR3-PerCP/Cy5.5 (Biolegend, Tokyo, Japan), and NKG2D-APC (Biolegend, Tokyo, Japan), similar to our previous study [2]. After fixation in 0.5% paraformaldehyde, samples were analyzed by flow cytometry (BD LSRII, BD Biosciences) within 24 hours of fixation. Data were analyzed with BD FACS Diva software (BD Biosciences, Franklin Lake, NJ).

### Protein analysis

Interleukin (IL)-1β, IL-2, IL-4, IL-5, IL-8, IL-10, IL-12p70, IL-13, IFN-γ, and TNF-α protein in the cell supernatants were analyzed with a Meso Scale Discovery Th1/Th2 10-plex tissue culture kit (Meso Scale Discovery, Gaithersburg, MD). Granzyme B and perforin levels were assessed in separate ELISA assays using commercially available kits (Diaclone ELISA kits (Gen-Probe, San Diego, CA, USA) according to the vendor’s instruction. Control experiments were conducted in which cytokine standards supplied with the ELISA kit were mixed with DEP and assessed for cytokine levels to assure no interference of DEP with the analysis (data not shown).

### Gene expression

Gene expression of granzyme B and perforin was measured via real-time quantitative PCR as previously published [52] and normalized to the reference gene 18-S.

### Cell-mediated cytotoxicity assay

After NK stimulation with pI:C, DEP, or pI:C+DEP for 18–20 hours, NK cells were analyzed using the 7-AAD/CFSE Cell-Mediated Cytotoxicity Assay Kit (Cayman Chemical Company, Ann Arbor, MI, USA) according to the supplier’s instructions. The human erythromyeloblastoid leukemia cell line K562 was used as target cells and was added to the stimulated NK cells (ratio of NK:target cells= 5:1) for 3 hrs.

### Data analysis

Differences between treatment and control were assessed with Wilcoxon Signed Rank Test. Effect of DEP on pI:C induced changes were tested with paired nonparametric t test. p<0.05 was considered as statistically significant. N=9 for surface marker, cytokine release, and gene expression and n=5 for the cytotoxicity assay.

## Abbreviations

BMI: Body mass index; CD: Cluster of differentiation; DEP: Diesel exhaust particles; IFN: Interferon; IL: Interleukin; MIC: MHC class I polypeptide-related sequence; NK: Natural killer; PBMC: Peripheral blood mononuclear cell; pI:C: Polyriboinosinic acid-polyribocytidylic acid; TNF: Tumor necrosis factor; ULBP: UL16-binding proteins.

## Competing interests

The authors have no conflicts of interest to disclose.

## Authors’ contributions

LM: conduction of cytotoxicity assay, data analysis, data organization, statistical analysis, manuscript preparation. CVEC: study design, sample processing and analysis, data organization, analysis, and manuscript preparation. MWH: conduction of cytotoxicity assay. TLN: Manuscript preparation. IJ: Study P.I., design and oversight of study design, and manuscript revision. All authors read and approved the final manuscript.
